# Triglyceride-Glucose Index for the Diagnosis of Metabolic Syndrome: A Cross-Sectional Study of 298,652 Individuals Receiving a Health Check-Up in China

**DOI:** 10.1155/2022/3583603

**Published:** 2022-06-29

**Authors:** Mingfei Jiang, Xiaoran Li, Huan Wu, Fan Su, Lei Cao, Xia Ren, Jian Hu, Grace Tatenda, Mingjia Cheng, Yufeng Wen

**Affiliations:** ^1^Department of Diagnostics, School of Clinical Medicine, Wannan Medical College, Wuhu 214002, Anhui, China; ^2^Department of Radiology, Nanjing Gaochun People's Hospital, Nanjing 211300, Jiangsu, China; ^3^Department of Health and Quarantine, School of Laboratory Medicine, Wannan Medical College, Wuhu 214002, AnHui, China; ^4^Department of Prevention Medical, School of Public Health, Wannan Medical College, Wuhu 214002, AnHui, China

## Abstract

**Objective:**

We herein aim to explore the relationship between the triglyceride-glucose (TyG) index and metabolic syndrome (MS).

**Methods:**

We enrolled 298,652 individuals with an average age of 47.08 ± 12.94 years and who underwent health check-ups at the First Affiliated Hospital of Wuhu Wannan Medical College in this cross-sectional study from 2014 to 2016. We enlisted 125,025 women (41.86%) and 173,627 men (58.14%). The survey information included a questionnaire survey, a physical examination, and a laboratory examination.

**Results:**

The prevalence of MS increased gradually in the TyG-index subgroups (*Q*1, TyG <8.30; *Q*2, 8.30≤ TyG <8.83; and *Q*3, TyG ≥8.83). We noted significant differences in hypertension, hyperlipidemia, hyperglycemia, sex, age, body mass index (BMI), smoking and drinking habits, and estimated glomerular filtration rate between the TyG-index subgroups. Multiclass logistic regression analysis showed that the group with TyG <8.30 was the reference group, and the 8.30≤ TyG <8.83 and the TyG ≥8.83 groups exhibited a higher TyG index with MS, and a lower TyG index without MS disease. In the linear curve analysis of the TyG index and MS components, BMI, systolic blood pressure, and diastolic blood pressure showed upward trends, while high-density lipoprotein cholesterol showed no obvious trend in the TyG index at a range of 7.8–11.0. Receiver operating characteristic analysis was used to evaluate the predictive value of the TyG index, triglycerides, and fasting blood glucose for MS, and we found that the area under the TyG index curve was the largest (AUC = 0.89).

**Conclusion:**

There were associations between the TyG index and MS and its components, and the TyG index is therefore of great value in the early diagnosis of MS.

## 1. Introduction

Metabolic syndrome (MS) and its components are important risk factors for cardiovascular diseases (CVDs) [1–3]. The components of MS—such as overweight or obesity, hypertension, hyperglycemia, hyperlipidemia, and atherosclerosis and stroke—are independently or jointly related [4–6]. Investigators reported that the risk of atherosclerosis in individuals with MS was twice as high as in those without MS [7], and other studies revealed that MS patients possessed a higher incidence of CVDs, especially with respect to ischemic stroke [8].

Insulin resistance (IR) is the principal feature of MS [9–11], and it can be used to independently predict the development of CVDs [12–14]. The TyG index is calculated using fasting blood glucose (FBG) and triglyceride (TG) levels and is considered to be a diagnostic index of IR that is reliable and relatively simple [15, 16]. Lu et al. demonstrated that patients with stroke exhibited high TyG index levels [17], and subsequent studies revealed that the TyG index of patients with carotid atherosclerosis was increased [18]. In addition, the TyG index combined with other biomarkers is often used to assess CVDs [19].

Although the relationship between MS and vascular diseases—as well as the relationships among hypertension, dyslipidemia, diabetes, and the TyG index—has been substantiated [20–22], there is no published report on the relationship between the TyG index and MS. A predictive relationship between the TyG index and MS or its components would therefore be of great value in the early diagnosis of MS.

## 2. Methods

### 2.1. Study Subjects

This cross-sectional study was based on 432,430 individuals who underwent health check-ups from 2011 to 2016 at the Physical Examination Center of the First Affiliated Hospital of Wannan Medical College, Wuhu, China. Our inclusion criteria were (1) 18–80 years of age; and (2) no presence of tumors or brain diseases. Exclusion criteria were (1) a lack of information on TG, FBG, high-density lipoprotein cholesterol (HDL-C), low-density lipoprotein cholesterol (LDL-C), total cholesterol (TC), systolic blood pressure (SBP), diastolic blood pressure (DBP), and body mass index (BMI); and (2) individuals with a history of tumors, brain diseases, severe CVDs, or severe infections. A total of 133,778 subjects were thereby excluded and 298,652 subjects (125,025 women (41.86%) and 173,627 men (58.14%)) were included. The mean age of our participants was 47.08 ± 12.94 years, and all completed questionnaire surveys, physical examinations, and laboratory measurements. This study was approved by the Ethics Committee of Wannan Medical College and followed the guidelines of the World Medical Association's Declaration of Helsinki. Informed verbal consent was obtained from all recruited participants prior to our investigation.

### 2.2. Questionnaire Surveys

Professional statisticians conducted standard questionnaire surveys and recorded baseline information on subjects. Demographic characteristics included age, sex, and educational background; behavioral characteristics included smoking (0 was defined as never smoking; 1 as occasionally smoking; 2 as frequently smoking), drinking (0 was defined as never drinking; 1 as occasionally drinking; 2 as frequently drinking); and history of disease and surgery included CVDs, severe infections, tumors, and major operations.

### 2.3. Physical Examinations

Professional clinicians perform physical examinations. A check-list included height, weight, and blood pressure. The height was measured barefoot and was accurate to 0.01 m, and the weight was measured wearing the lightest clothes and was accurate to 0.1 kg. BMI was calculated by weight (kg)/height square (m^2^), accurate to 0.01 kg/m^2^. After resting for at least 5 minutes, blood pressures were measured three times using a Mercury sphygmomanometer at 1-minute intervals. The average systolic and diastolic blood pressures were calculated and used for subsequent analysis.

### 2.4. Laboratory Measurements

After 12 hours of fasting, 5 mL of venous blood was collected from participants. The parameters assayed were FBG, TG, HDL-C, TC, LDL-C, and eGFR using an automatic analyzer. All biochemical assays were performed by laboratory technicians at the hospital.

### 2.5. Definitions

MS was defined as (1) overweight and/or obese (BMI ≥25 kg/m^2^), (2) hyperglycemic (FBG ≥6.1 mmol/L and/or a 2-h postprandial blood glucose ≥7.8 mmol/L, or under treatment with antihyperglycemic drugs); (3) hypertensive (SBP ≥140 mm Hg, and/or DBP ≥90 mm Hg, or under treatment with antihypertensive drugs); and (4) hyperlipidemic (TG ≥ 1.7 mmol/L, and/or HDL_C < 0.9 mmol/L in males, HDL-C < 1.0 mmol/L in females) [23].

Frequent smokers are those who have smoked continuously for 6 months or more, one or more cigarettes per day; occasional smoking is continuous smoking for 6 months, no less than four times per week, and no more than once per day on average [24].

Frequent drinking: the daily drinking amount of 56-degree white wine is not less than 50 ml, and the drinking frequency is more than 5 times per week. Occasional drinking: the drinking amount is less than 50 ml per day and the number of drinks is less than 5 times per week [25].

### 2.6. Statistical Analysis

We employed simple descriptive statistics and expressed continuous variables as mean ± standard deviation, and categorical variables as frequency (%). The demographic characteristics and biochemical indicators of groups that we divided by triquantile were compared by analysis of variance (*Q*1, TyG <8.30; *Q*2, 8.30≤ TyG <8.83; *Q*3, TyG ≥8.83), with *Q*1 as the reference group. We used a multiclass logistic regression model to analyze the relationship between TyG index and common chronic diseases; adjusted for possible confounding factors such as age, sex, smoking, drinking, BMI, and estimated glomerular filtration rate (eGFR); and provided an odds ratio (OR) and 95% confidence interval (CI). Three multivariate logistic regression models were constructed to identify the associations between TyG index and MS. Model I was unadjusted; model II was adjusted for age, sex, BMI, smoking, and drinking; and model III was adjusted for SBP, DBP, TC, LDL-C, HDL-C, TG, FBG, and eGFR and based upon model II. We employed *R* 2.4.1 and used the mgcv and ggplot packages to explore the generalized linear curve for the TyG index and MS components (the TyG index was calculated using FBG and TG, and thus their relationships with FBG and TG were not explored). Receiver operating characteristic (ROC) curves for the TyG index, FBG, and TG were implemented to evaluate the best threshold for MS and the area under the curve (AUC).

## 3. Results

### 3.1. Demographic Characteristics and Biochemical Indicators of TyG-Index Subgroups

Our results revealed that the prevalence of MS gradually increased in the three groups (*Q*1 was 0.26%, *Q*2 was 1.48%, and *Q*3 was 11.01%). Hypertension, hyperlipidemia, and hyperglycemia were statistically significant among the three groups with respect to the TyG index. In addition, there were significant differences in other baseline characteristics of the group—including sex, age, BMI, smoking and drinking habits, and eGFR (Table 1).

### 3.2. Relationship between Components of MS and the TyG Index

Adjusting those indicators with significant differences among the TyG-index subgroups and based on the analysis of the multiclass logistic regression model, we found that the Q1 group was the reference group; and that the TyG index in individuals with MS in the Q2 and Q3 groups was elevated, while the TyG index was lower without MS (OR = 2.68, 95% CI = 2.35–3.07; OR = 16.56, 95% CI = 14.59–18.79). In model I of the multivariate logistic regression models, when the TyG index increased by 1 unit, the prevalence of MS increased by 5.57 units; in model II, when the TyG index increased 1 unit, the prevalence of MS increased by 5.46 units; in model III, when the TyG index increased 1 unit, the prevalence of MS increased by 34.09 units (Tables 2–4).

**Figure 1:**
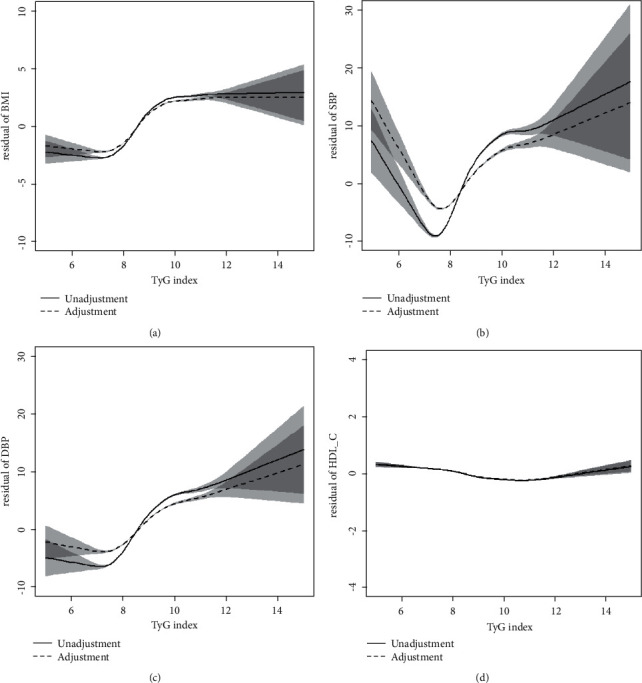
Relationship between TyG index and MS components. The *X*-axis represents the TyG index; the *Y*-axis shows the residual of the MS components. Solid line, unadjusted; dotted line: adjusted for age, sex, BMI, smoking, drinking, SBP, DBP, TC, HDL-C, LDL-C, TG, FBG, and eGFR (when used as the longitudinal coordinate, it was not adjusted). (a) Correlation of BMI with TyG index. (b) Correlation of SBP with TyG index. (c) Correlation of DBP with TyG index. (d) Correlation of HDL-C with TyG index. TyG index was calculated using the FBG and TG indices, and thus their relationship with FBG and TG was not discussed further. TyG, triglyceride-glucose; BMI, body mass index; SBP, systolic blood pressure; DBP, diastolic blood pressure; HDL-C, high-density lipoprotein cholesterol.

**Figure 2:**
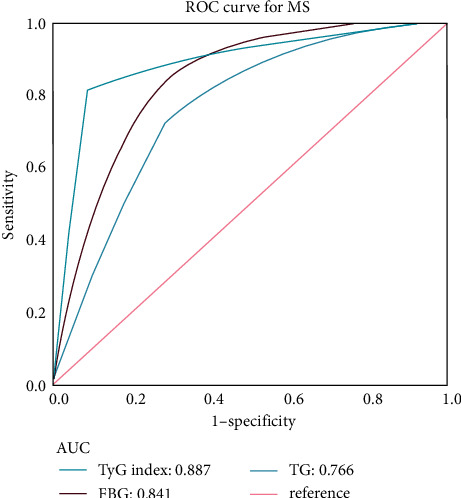
Receiver operating characteristic curve analyses for predicting MS. AUC, area under the curve; TyG, triglyceride-glucose; FBG, fasting blood glucose; TG, triglyceride.

After adjusting for potential risk factors, the relationship between TyG and MS components changed with the TyG index. The TyG index ranged from 7.8 to 11.0; BMI, SBP, and DBP showed an upward trend; and HDL-C exhibited no obvious fluctuation trend. When we employed ROC analysis to evaluate the predictive value of the TyG index, TG, and FBG with respect to MS, we found that the AUC of the TyG index was the largest at 0.89 (with an optimal threshold of 8.85, sensitivity of 0.81, and specificity of 0.91); we thus regarded this as an effective diagnostic index for MS (Figures 1 and 2 and Table 5).

## 4. Discussion

In this study, we demonstrated differences in the TyG index between populations with MS and those without MS, and there were correlations between MS components and the TyG index. These results are consistent with IR being a contributing factor in the development of MS [26–28]. The TyG index is a reliable and simple diagnostic indicator of insulin resistance [29, 30]. When the body is in the IR state, the PI3K pathway is affected, with the MAPK pathway unaffected; and the balance between the two pathways is therefore disrupted [31, 32]. Inhibition of the PI3K pathway leads to a reduction in the production of NO in endothelial cells, which in turn reduces ED and glucose transporters and results in a reduction in glucose uptake [33]. In contrast, the MAKP pathway continues to produce endothelin-1 that stimulates vasoconstriction and accelerates the division of fat cells, causing MS [34, 35].

This study revealed that the TyG index was in the range of 7.8–11.0; that BMI, SBP, and DBP showed an upward trend; and that HDL-C did not fluctuate significantly. Bovolini et al. found that abdominal obesity was a form of obesity primarily related to IR, producing a clinical manifestation of increased waist circumference. Obesity or fatty tissue increases may also lead to a lack of blood supply and oxygen [26], and hypoxia is the basis for tissue necrosis and macrophage infiltration. Free fatty acids produced by fat cells are biologically active metabolites that can damage pancreatic *β*-cell functioning, and the fibrinogen and PAI-1 produced can exacerbate thrombosis [36, 37]. An elevation in fibrinogen levels indicates that the body is in an acute inflammatory phase, and PAI-1 is regarded as a marker of abnormal fibrinolysis and atherosclerotic thrombosis [38]. The TyG index exhibited an upward trajectory along with SBP and DBP, emulating the results of the Quesada study [39]. In addition, Antonio-Villa et al. stated that IR exerted a stronger effect on the risk of hypertension in women than in men [40]. IR increases the renal reabsorption of sodium directly or indirectly, stimulating endothelial cells to produce profibrotic transforming factor *β*1 and increase vascular adhesion molecules [33, 41]. Excessive sodium is then the cause of the increase in blood pressure. By disrupting the surface of the endothelial glycosides, this accelerates the changes in vascular endothelial cells, leading to invasion by inflammatory cells and diminishing blood-vessel density. Second, IR stimulates pro-opiomelanocortin (POMC)-releasing neurons in the arcuate nucleus of the hypothalamus, and activates glutamatergic neurons and neurons that express melanocortin 4 receptor (MC4R) in the paraventricular nucleus; thus enhancing the overall activity of the sympathetic nervous system [42, 43]. The vasodilatory effect observed in hypertensive patients is thus impaired, with sympathetic nerve activation three times that in normal, unaffected individuals [44]. Lin et al. uncovered IR as an alarming cause of impaired glucose metabolism and dyslipidemia in the body [45], and Feng indicated that IR was directly correlated with triglycerides and total cholesterol in Chinese patients [46]. Previous studies have paid more attention to the relationship between the ratios of triglyceride to HDL-C and IR, and less direct attention to HDL-C [47, 48]. Herein, we found that HDL-C did not change significantly, and the reason for this is unclear. Some unknown underlying mechanism(s) may exist, and this needs to be further examined in the future. Yu et al. pointed out that the TyG index is more suitable for identifying individuals with unhealthy metabolism in the Chinese adult population. The optimal cutoff value of the TyG index in the metabolically unhealthy male population was 8.81, and the best cutoff value in the female population was 8.71 [49]. Lin et al. showed that the TyG index rather than the blood leukocyte index may have the strongest predictive value for the development of MetS within 5 years. The best cutoff value for the prediction of MetS events by the TyG index based on the Youden index was 8.52 [50]. Son et al. found that the critical point of the TyG index for predicting the prevalence of MetS was 8.718, and the critical points for predicting event MetS was 8.518 [51]. In the present study the optimal cutoff value for the TyG index in predicting MS as determined by ROC curve analysis was 8.85, which was similar to the best cutoff value observed in other studies.

### 4.1. Limitations

First, this was a cross-sectional study, and we therefore could not establish a causal relationship directly based on our results. Additional prospective studies are needed to explore the relationship between the TyG index and MS. Second, fasting blood glucose and triglycerides change over time, and we only collected baseline values and could not conduct time-correlation analysis. Finally, the mechanism(s) underlying the insignificant and fluctuating trend for HDL-C remains arcane. The health check-up population varies from the general population, so the representativeness of this conclusion is limited. However, this was an epidemiological survey based on a large sample size, and the results that we obtained possessed extremely high reliability and validity.

## 5. Conclusion

We concluded that there was a relationship between the TyG index and MS and its components. The TyG index manifested great value in the early diagnosis of MS, which suggested that in a population with a significantly increased TyG index, it is necessary to identify and control multiple metabolically related risk factors.

## Figures and Tables

**Table 1:** Comparison of the demographic characteristics and biochemical indices among the TyG-index subgroups.

Variables	Total	*Q*1 (TyG <8.30)	*Q*2 (8.30≤ TyG <8.83)	*Q*3 (TyG ≥8.83)	*χ* ^2^	*P*
Sex					22142.40	<0.001
Female	125,025 (41.86)	58,576 (58.83)	40,638 (40.82)	25,811 (25.93)		
Male	173,627 (58.14)	40,986 (41.17)	58,911 (59.18)	73,730 (74.07)		
Age (years)					13289.19	<0.001
≤45	47.08 ± 12.94	61,491 (61.76)	43,082 (43.28)	34,123 (34.28)		
46–69		34,585 (34.74)	50,199 (50.43)	58,795 (59.07)		
>70		3486 (3.50)	6268 (6.30)	6623 (6.65)		
BMI (kg/m^2^)					36285.17	<0.001
<25	23.80 ± 3.24	85,072 (85.45)	67,420 (67.73)	44,813 (45.02)		
≥25		14,490 (14.55)	32,129 (32.27)	54,728 (54.98)		
Drinking					9507.51	<0.001
0	198,151 (66.35)	78,283 (78.63)	66,750 (67.05)	53,118 (53.36)		
1	28,633 (9.59)	4814 (4.84)	8736 (8.78)	15,083 (15.15)		
2	71,868 (24.06)	16,465 (16.54)	24,063 (24.17)	31,340 (31.48)		
Smoking					5481.96	<0.001
0	217,146 (72.71)	82,391 (82.75)	72,850 (73.18)	61,905 (62.19)		
1	64,904 (21.73)	12,845 (12.90)	20,985 (21.08)	31,074 (31.22)		
2	16,602 (5.56)	4326 (4.35)	5714 (5.74)	6562 (6.59)		
Hypertension					14195.33	<0.001
No	243,611 (81.57)	91,073 (91.47)	82,095 (82.47)	70,443 (70.77)		
Yes	55,041 (18.43)	8489 (8.53)	17,454 (17.53)	29,098 (29.23)		
Hyperlipidemia					25047.02	<0.001
No	272,388 (91.21)	98,010 (98.44)	96,387 (96.82)	77,991 (78.35)		
Yes	26,264 (8.79)	1552 (1.56)	3162 (3.18)	21,550 (21.65)		
Hyperglycemia					24470.19	<0.001
No	263,745 (88.31)	97,326 (97.75)	91,537 (91.95)	74,882 (75.23)		
Yes	34,907 (11.69)	2236 (2.25)	8012 (8.05)	24,659 (24.77)		
MS					14138.52	<0.001
No	285,967 (95.75)	99,305 (99.74)	98,077 (98.52)	88,585 (88.99)		
Yes	12,685 (4.25)	257 (0.26)	1472 (1.48)	10,956 (11.01)		
eGFR					441.35	<0.001
<60	58,854 (19.71)	20,884 (20.98)	20,819 (20.91)	17,151 (17.23)		
≥60	239,798 (80.29)	78,678 (79.02)	78,730 (79.09)	82,390 (82.77)		
Total	298,652	99,562	99,549	99,541		

For drinking, 0 = never drinking, 1 = occasional drinking, and 2 = frequent drinking. For smoking, 0 = never smoking, 1 = occasional smoking, and 2 = frequent smoking. TyG, triglyceride-glucose; BMI, body mass index; eGFR, estimated glomerular filtration rate; MS, metabolic syndrome.

**Table 2:** Multiclass logistic regression among the TyG-index subgroups.

Variables	*Q*1 (TyG <8.30)	*P*	*Q*2 (8.30≤ TyG <8.83)	*P*	*Q*3 (TyG ≥8.83)	*P*
	OR (95% CI)		OR (95% CI)		OR (95% CI)	
Hypertension	1.00		2.28 (2.22–2.34)	<0.001	4.43 (4.32–4.55)	<0.001
Hyperglycemia	1.00		3.80 (3.63–4.00)	<0.001	14.33 (13.71–14.98)	<0.001
Hyperlipidemia	1.00		2.07 (1.95–2.20)	<0.001	17.43 (16.54–18.37)	<0.001
MS	1.00		5.80 (5.08–6.62)	<0.001	7.79 (7.22–8.10)	<0.001
Hypertension^*∗*^	1.00		1.30 (1.26–1.33)	<0.001	1.86 (1.80–1.91)	<0.001
Hyperglycemia^*∗*^	1.00		2.51 (2.39–2.64)	<0.001	8.17 (7.80–8.56)	<0.001
Hyperlipidemia^*∗*^	1.00		2.77 (2.60–2.95)	<0.001	2.90 (2.73–3.09)	<0.001
MS^*∗*^	1.00		2.68 (2.35–3.07)	<0.001	16.56 (14.59–18.79)	<0.001

^*∗*^Adjusted for age, sex, BMI, smoking, drinking, and eGFR. TyG, triglyceride-glucose; MS, metabolic syndrome; OR, odds ratio; CI, confidence interval.

**Table 3:** Comparison of the demographic characteristics and biochemical indices between MS subgroups.

Variables	Non-MS (*n* = 285,967)	MS (*n* = 12,685)	*χ* ^2^/*t*	*P*
TyG index	8.58 ± 0.61	9.39 ± 0.59	151.08	<0.001
Sex			235.10	<0.001
Female	118,881 (41.57)	6144 (48.44)		
Male	167,086 (58.43)	6541 (51.56)		
Age (years)				
≤45	137,115 (47.95)	1581 (12.46)	7269.66	<0.001
46–69	134,442 (47.01)	9137 (72.03)		
>70	144,10 (5.04)	1967 (15.51)		
BMI (kg/m^2^)			22626.06	<0.001
<25	196,774 (68.81)	531 (4.19)		
≥25	89,193 (31.19)	12,154 (95.81)		
Drinking			45.45	<0.001
0	189,750 (66.35)	8401 (66.23)		
1	26,925 (9.42)	1708 (13.46)		
2	69,292 (24.23)	2576 (20.31)		
Smoking			166.64	<0.001
0	207,337 (72.50)	9809 (77.33)		
1	62,489 (21.85)	2415 (19.04)		
2	16,141 (5.64)	461 (3.63)		
Hypertension			44618.30	<0.001
No	242,290 (84.73)	1321 (10.41)		
Yes	43,677 (15.27)	11,364 (89.59)		
Hyperlipidemia			17664.97	<0.001
No	264,967 (92.66)	7421 (58.50)		
Yes	21,000 (7.34)	5264 (41.50)		
Hyperglycemia			61940.62	<0.001
No	261,355 (91.39)	2390 (18.84)		
Yes	24,612 (8.61)	10,295 (81.16)		
eGFR			1884.72	<0.001
<60	54,451 (19.04)	4403 (34.71)		
≥60	231,516 (80.96)	8282 (65.29)		
SBP (mm Hg)	118.00 ± 15.95	143.20 ± 15.80	174.28	<0.001
DBP (mm Hg)	76.90 ± 9.63	89.22 ± 9.83	138.23	<0.001
TG (mmol/L)	1.52 ± 1.19	2.54 ± 1.85	61.88	<0.001
TC (mmol/L)	4.61 ± 0.89	5.09 ± 1.00	53.60	<0.001
HDL_C (mmol/L)	1.38 ± 0.35	1.26 ± 0.29	41.42	<0.001
LDL_C (mmol/L)	2.55 ± 0.77	2.73 ± 0.89	21.46	<0.001
FBG (mmol/L)	5.31 ± 1.01	7.15 ± 2.02	101.68	<0.001

MS, metabolic syndrome; TyG, triglyceride-glucose; BMI, body mass index; eGFR, estimated glomerular filtration rate; SBP, systolic blood pressure; DBP, diastolic blood pressure; TG, triglyceride; TC, total cholesterol; HDL-C, high-density lipoprotein cholesterol; LDL-C, low-density lipoprotein cholesterol; FBG, fasting blood glucose.

**Table 4:** The relationship between TyG index and MS across the three models.

Model	*β*	S.E.	Wald *χ*^2^	*P*	OR	95% CI
I	1.72	0.01	15,626.11	<0.001	5.57	5.42–5.72
II	1.70	0.02	9153.95	<0.001	5.46	5.28–5.66
III	3.70	0.07	2577.26	<0.001	34.09	31.47–36.93

Model I, adjusted for no confounding factors. Model II, adjusted for age, sex, BMI, smoking, drinking. Model III, adjusted for SBP, DBP, TC, LDL-C, HDL-C, TG, FBG, and eGFR on the basis of Model II. *β*, coefficient; S.E., standard error; OR, odds ratio; CI, confidence interval.

**Table 5:** Areas under the receiver operating characteristic curves for TyG index, FBG, and TG in identifying MS.

Variables	AUC	95% CI lower	95% CI upper	Optimal threshold	Sensitivity	Specificity	*P*
TyG index	0.89	0.88	0.89	8.85	0.81	0.91	<0.001
TG	0.77	0.76	0.77	1.67	0.73	0.71	<0.001
FBG	0.84	0.84	0.84	6.10	0.86	0.70	<0.001

TyG, triglyceride-glucose; TG, triglyceride; FBG, fasting blood glucose; AUC, area under the ROC curve; CI, confidence interval.

## Data Availability

No data were used to support this study.
